# Comparison of commonly used software pipelines for analyzing fungal metabarcoding data

**DOI:** 10.1186/s12864-024-11001-x

**Published:** 2024-11-14

**Authors:** Theresa Rzehak, Nadine Praeg, Giulio Galla, Julia Seeber, Heidi Christine Hauffe, Paul Illmer

**Affiliations:** 1 Department of Microbiology, Universität Innsbruck, Innsbruck, Austria; 2 Conservation Genomics Research Unit, Research and Innovation Centre, Fondazione Edmund Mach, S. Michele all’Adige, Italy; 3 Institute for Alpine Environment, EURAC Research, Bolzano, Italy; 4 Department of Ecology, Universität Innsbruck, Innsbruck, Austria; 5 National Biodiversity Future Center (NBFC), S.c.a.r.l., Palermo, Italy

**Keywords:** Soil fungi, Animal microbiota, Internal transcribed spacer (ITS), Bioinformatics, DADA2, Mothur

## Abstract

**Background:**

Metabarcoding targeting the internal transcribed spacer (ITS) region is commonly used to characterize fungal communities of various environments. Given their size and complexity, raw ITS sequences are necessarily processed and quality-filtered with bioinformatic pipelines. However, such pipelines are not yet standardized, especially for fungal communities, and those available may produce contrasting results. While some pipelines cluster sequences based on a specified percentage of base pair similarity into operational taxonomic units (OTUs), others utilize denoising techniques to infer amplicon sequencing variants (ASVs). While ASVs are now considered a more accurate representation of taxonomic diversity for prokaryote communities based on 16S rRNA amplicon sequencing, the applicability of this method for fungal ITS sequences is still debated.

**Results:**

Here we compared the performance of two commonly used pipelines DADA2 (inferring ASVs) and mothur (clustering OTUs) on fungal metabarcoding sequences originating from two different environmental sample types (fresh bovine feces and pasture soil). At a 99% OTU similarity threshold, mothur consistently identified a higher fungal richness compared to DADA2. In addition, mothur generated homogenous relative abundances across multiple technical replicates (*n* = 18), while DADA2 results for the same replicates were highly heterogeneous.

**Conclusions:**

Our study highlights a potential pipeline-associated bias in fungal metabarcoding data analysis of environmental samples. Based on the homogeneity of relative abundances across replicates and the capacity to detect OTUs/ASVs, we suggest using OTU clustering with a similarity of 97% as the most appropriate option for processing fungal metabarcoding data.

**Supplementary Information:**

The online version contains supplementary material available at 10.1186/s12864-024-11001-x.

## Background

Fungi comprise a clade of eukaryotes with diverse life forms. They are colonizing every habitat on the planet, utilizing all substrates including other living organisms [1]. Remarkably, over 90% of all known fungal species inhabit soil [2], where they are known to play key roles in nutrient cycling, impacting environmental physicochemical properties, and the health of other eukaryotes [3]. Even so, it has been estimated that more than 90% of fungal taxa have not yet been discovered [4, 5]. Traditionally, fungal taxonomy has relied on laboratory cultures and the identification of fruiting bodies, but this is a relatively inefficient taxonomic method due to the diverse morphological and developmental features of fungi, especially as not all taxa are culturable. In recent years, high-throughput-sequencing (HTS) methods have resulted in an exponential increase in the detection of new fungal species from various environments and matrices, including living organisms [6]. With this culture-independent technique, individual fungal species can be identified from sequences of DNA or RNA extracted from various sample types [6], allowing the comparison of fungal taxa and communities between various environments [7]. Commonly, the nuclear 18S (small subunit, SSU) and 28S (large subunit, LSU) ribosomal RNA (rRNA) genes as well as the internal transcribed spacer (ITS) region have been the focus of such studies [7]. Among these, the ITS region, especially the ITS subregions ITS1 and ITS2, have proven the most useful loci for fungi identification due to their high interspecific variation [8].

Prior to the statistical analysis of metabarcoding results, the generated sequences must undergo sequence processing (e.g. clustering, classification) and quality control. Various software pipelines have been developed and made freely available, each with a diverse set of tools; however, since these have mainly been developed for prokaryote 16S rRNA sequences, they are not all considered equally suitable for fungal ITS analysis [1]. Among the most cited sequence analysis pipelines are mothur [9] and DADA2 [10]. Mothur is a free, open-source software, which can be executed in the command line [9]. It incorporates OTU-clustering by the robust and memory-efficient OptiClust algorithm [11]. Mothur provides a fully transparent workflow, allowing the user to track all steps during sequence processing. All commands can be specified by the user, if needed. Although originally designed for prokaryote (16S rRNA) amplicon sequencing [7], mothur is also commonly used for other HTS studies with various barcode markers. DADA2 is also an open-source software, available as an R package. It includes a full sequencing workflow and applies the most frequently used ASV-construction method, allowing accurate and high-resolution community construction [10]. A readily available workflow is provided and it is applicable for any target locus (short and long reads), although the application to certain loci is debated [7]. The first steps of sequence processing in both of the above software pipelines include primer trimming, and removal (or ‘filtering’) of poor-quality reads (e.g. those with low-quality scores, high number of homopolymers, ambiguous bases), non-target reads and the Illumina-specific merging of paired-end reads (in DADA2, the latter is carried out at a later step of the workflow). Finally, true biological variation is distinguished from unwanted, sequencing error-induced variation, sequences are compared to available databases, and taxa are identified. For fungi, given the limited knowledge of intraspecific variation, similar sequences are often aggregated into species-level operational taxonomic units (OTUs) to avoid overestimating species richness. Among several *de novo* clustering approaches, the OptiClust algorithm, implemented in the mothur pipeline, produces high-quality OTU assignments at low computational load while simultaneously evaluating clustering quality using the Matthews correlation coefficient (MCC) [11]. Such clustering implies the setting of a ‘sequence similarity threshold’, which is usually a compromise between the highest possible differentiation between species, and correction for sequencing errors [7]. Although a 97% similarity threshold is often used, it has been noted this may result in an underestimation of the true number of observed fungal species [12, 13]. Other studies highlight that a higher threshold (e.g. 99%) might be more appropriate for this OTU clustering method [7]. Instead, DADA2 generates amplicon sequence variants (ASVs) based on an error model calculation, and assigns sequences with a minimum of one nucleotide difference to separate taxa, or removes these as potential noise [10]. However, it has been suggested that, given the high levels of intragenomic variation of fungal ITS, the ASV approach for fungal ITS data may inflate the number of observed species (observed non-identical sequences) [14]; hence, the applicability of ASV approaches for the fungal ITS region is highly debated [1, 15–18].

Because initial data processing can impact the results and their interpretation, researchers must constantly evaluate the available bioinformatic opportunities in a fast-evolving field; therefore, studies comparing different pipelines are useful for promoting efficient workflows. Not only do the currently available software pipelines vary in their applicability to fungal metabarcoding data [1, 15], but pipeline comparison studies have tended to focus on mock communities [19], and simulated datasets [20], both of which suffer from oversimplification. In fact, no pipeline developed thus far has performed satisfactorily when tested on fungal mock communities [7, 19]. Hence, testing the performance of different pipelines on fungal ITS datasets generated from complex field-collected environmental samples is of particular interest.

Here, we evaluated the performance of mothur using both 97% and 99% identity thresholds and DADA2 in analyzing fungal communities from two different field-collected sample types often used for targeted metagenomic studies: fresh bovine feces and pasture soil. For a set of 19 biological replicates (10 bovine feces and nine soil samples from different sample sites), we compared alpha and beta diversity generated by the three different pipelines. In addition, for a set of 36 technical replicates of both sample types (one biological sample each of bovine feces and soil, amplified 18 times each), we compared the basic read output, community composition, taxonomic classification, homogeneity of results among the replicates, and capacity of each pipeline to detect OTUs/ASVs. Furthermore, we examined the impact of different similarity thresholds for OTU clustering on fungal community results. To our knowledge, this is the first time a comparison of the performance of the OptiClust OTU clustering method has been compared to that of other pipelines for fungal metabarcoding data.

## Materials and methods

### Dataset

Fresh bovine fecal and pasture soil samples (in total 19 samples) were collected in June 2019 from an Alpine pasture in the Long-Term Social-Ecological Research Area (LTSER) Val Mazia/Matschertal (Province of Bolzano, Italy) as part of the EUREGIO project Microvalu (as described in refs. 18 and 19). These sample types were selected for testing the performance of the selected pipelines, as they represent two highly diverse fungal sources from this grassland ecosystem.

Three to four bovine fecal samples were collected from each of three different sites (approx. 500 m apart) at an elevation of 1500 m a.s.l. from freshly deposited cow pats. For each of the 10 samples (= biological replicates), about 50 g of feces were collected from three points on the pat, transferred to a sterile polypropylene tube and mixed thoroughly using a sterile spatula; the samples were then transported to the Fondazione E. Mach at 4 °C, and archived at -80 °C until further processing.

Bulk soil (Lithic Leptosol – World Reference Base for Soil Resources) was collected from three pastured grassland sites (approx. 500 m apart) at an elevation of 2500 m a.s.l. The vegetation cover was carefully removed with a shovel and soil was gathered from the upper mineral horizon at 12–20 cm soil depth (Ah horizon). Each bulk soil sample was composed of 10 subsamples (approx. 100 g each), which were combined into a composite soil sample. In total, nine soil samples (= biological replicates) were prepared, transported to the Universität Innsbruck at ambient temperature after a few hours, and processed following [21] and [22]. In brief, 100 mg of soil sieved at 1 mm from each biological replicate was suspended in 10 ml of sterile ¼ Ringer containing 0.01% (v/v) Tween^®^ 80 solution in a sterile polypropylene tube. The soil solution was shaken on an overhead shaker for 10 min at 90 rpm and treated in an ultrasonic bath for 1 min. The soil slurry was centrifuged at 10,000 x g for 15 min and the supernatant was discarded.

### DNA extraction, amplification and ITS2 sequencing

**Fig. 1:**
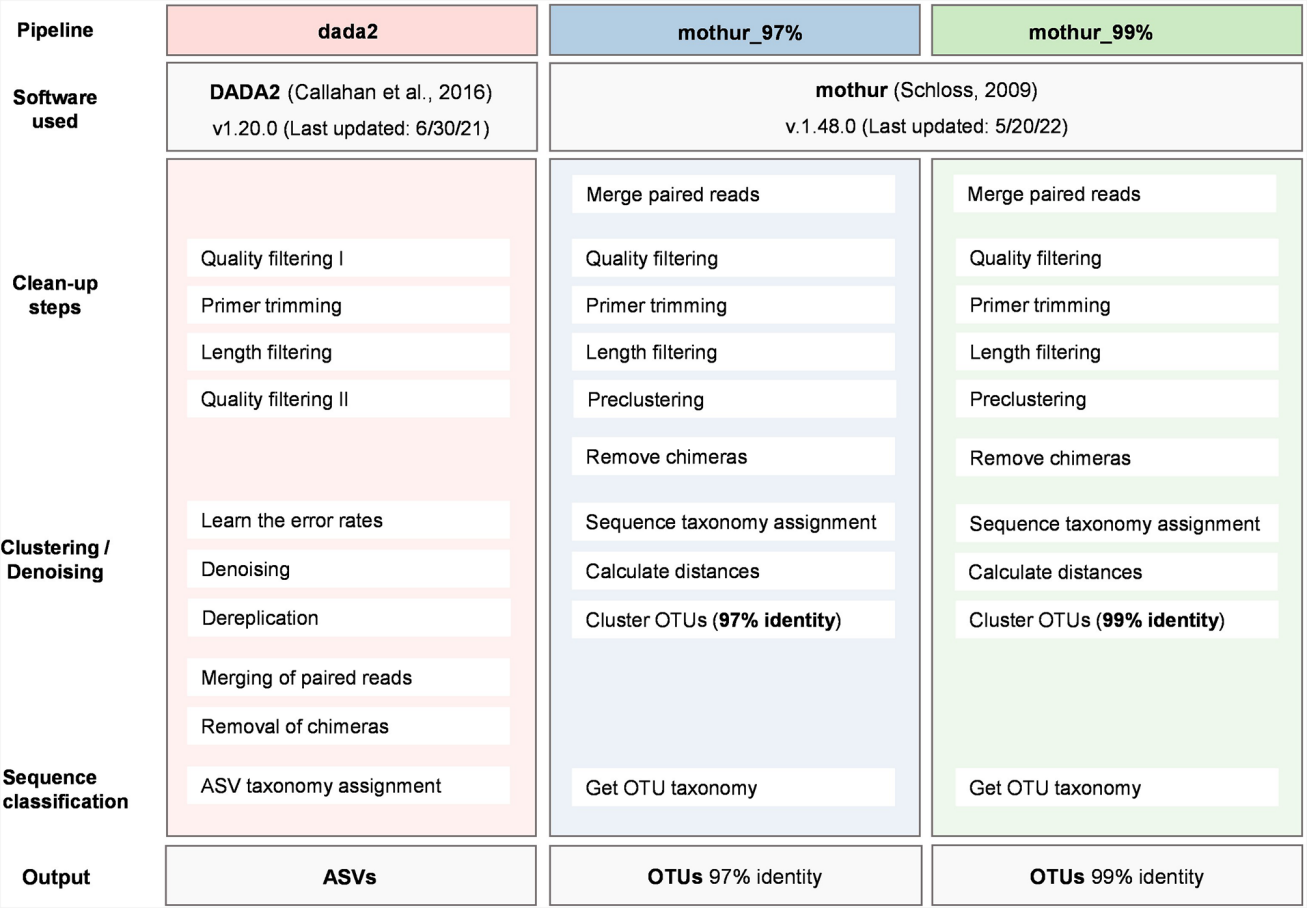
Schematic overview comparing the three bioinformatic downstream analysis pipelines (dada2, mothur_97%, mothur_99%) using two freely available software tools, mothur and DADA2. Each textbox represents the key steps within the respective pipeline

DNA was extracted from each of the nine soil and 10 bovine feces biological replicates using the NucleoSpin^®^ Soil kit (Macherey-Nagel, Germany) to allow direct comparison of microbiota of the different sample types [23] and following the manufacturer’s protocol, with minor modifications, i.e.: (i) homogenization time was doubled, (ii) buffer SL1 was used for the lysis step, and (iii) a volume of 50 µL of enhancer buffer (SX) was added to the sample during lysis. For whole DNA extraction, 70 mg of fecal matter and 30 mg of soil slurry were used as input biomass, respectively. Extraction controls, containing no sample material (lysis buffer only), were included to exclude contaminations in subsequent analyses. Purity and quantity of the DNA extracts were checked by UV/VIS spectrometry using a Spark^®^ multimode microplate reader (Tecan, Switzerland). The ITS2 region, which is recommended for fungal biodiversity studies [7, 24, 25] and is widely used [26–28], was selected for amplification. Our primers of choice were ITS4_ILL and gITS7_ILL [29, 30], of which both are among primers recommended for high-throughput identification of fungi [31] and result in high fungal coverage [1]. To generate technical replicates, one randomly selected biological replicate each of the bovine feces and soil samples was amplified 18 times. For the amplification of the fungal ITS2 region, 9 ng of extracted DNA was mixed with 1x FastStart High Fidelity Reaction Buffer (Roche Applied Science), 1.5 U of FastStart High Fidelity Enzyme Blend (Roche Applied Science) and primers ITS4_ILL / gITS7_ILL [29, 30] to a final concentration of 0.4 µM, resulting in 30 µL final PCR mix per replicate. PCR reactions were performed on a Veriti™ 96-Well Fast Thermal Cycler (Applied Biosystems, USA) using the following conditions: 3 min at 95 °C, followed by 31 cycles of 30 s at 95 °C, 30 s at 50 °C, 30 s at 72 °C, and a single final extension step of 7 min at 72 °C. Non-template controls (amplification controls), containing no DNA but amplification mix only, were included. Quality of the amplicons was checked by performing a high-resolution capillary electrophoresis using the QIAxcel Advanced System (QIAGEN). High-throughput sequencing was performed by the FEM Sequencing and Genotyping Platform (San Michele all’Adige, Italy) on an Illumina MiSeq Standard Flow Cell, using v3 chemistry and 300 bp paired-end reads and a minimum depth of 30,000 reads per sample. For sequence processing, we used two software platforms (DADA2 and mothur) and created the following three ‘pipelines’: DADA2, generating ASVs (hereafter ‘dada2 pipeline’); mothur, generating OTUs with a similarity threshold of 97% (‘mothur_97% pipeline’) or 99% (‘mothur_99% pipeline’), using default commands given by each of the software publishers (Fig. 1). The same quality filtering and taxonomy assignment settings were adopted in both pipelines to facilitate the comparison of results (see conditions below). Additionally, raw read processing was also conducted with the default settings or recommended standard operating procedures for quality filtering and taxonomy assignment of fungal reads. This allowed us to evaluate the results of each pipeline using the most commonly used approaches in microbial ecology. The applied settings and the results of this additional analysis are provided in the supplement (see Supplementary Information, Material & Methods and Results & Discussion sections). Raw sequencing data were deposited in the NCBI Sequence Read Archive (SRA) and are accessible under the BioProject ID: PRJNA1055419. Details of the samples are provided in the Supplementary Table (Supplementary Material 2).

### Bioinformatic downstream analysis generating ASVs – dada2 pipeline

In the dada2 pipeline, barcode free, paired-end reads of demultiplexed samples were processed following an ITS-specific adaptation of the 1.8 DADA2 tutorial workflow (https://benjjneb.github.io/dada2/ITS_workflow.html), using the DADA2 package [10] in R (v 4.2.0, 23). Primers were removed with Cutadapt [32] and, reads were quality filtered using the *filterAndtrim* function and the following settings (unified among pipelines): reads less than 100 bp in length, having ambiguous bases or ‘bad quality’ were discarded, where bad quality reads were defined as reads not passing the *filterAndtrim*-parameter maxEE = c(2,2). The settings maxN, minLen and maxEE were the specified optional arguments in the *filterAndtrim* function. Reads were not truncated to uniform lengths, to maintain length polymorphisms of the ITS region [17]. ASVs were generated using the DADA2 inference algorithm by the *learnErrors* and *dada* functions. Reads were merged (*mergePairs* function) and chimeric sequences were removed (*removeBimeraDenovo* function). Taxonomic classification was assigned (*assignTaxonomy* function) using the UNITE (v8.3) database [33] and the RDP Naive Bayesian Classifier algorithm [34]. The bootstrap cutoff for assignment was set to 80% for all pipelines.

### Bioinformatic downstream analysis generating OTUs – mothur_97% and mothur_99% pipelines

Fungal OTUs were constructed using mothur (v.1.48.0) following the MiSeq SOP (last access 6/10/22) [9]. Forward and reverse reads were merged using the *make.contigs* function setting the following parameters (unified among pipelines): maxambig = 0, maxee = 2, deltaq = 0. No read length truncation was performed for the reasons explained above. After primers were trimmed with the *trim.seq* function, sequences with less than 100 bp in length were discarded. Sequences were pre-clustered allowing a maximum of three base pair differences between reads; chimeric sequences were also removed. Sequences were classified with the *classify.seqs* function using the UNITE (v8.3) database [33] and applying the RDP Naive Bayesian Classifier algorithm [34]. The bootstrap cutoff for the taxonomy assignment was unified for all pipelines (set to 80%). OTUs were identified after calculating distances between sequences (*pairwise.seqs* function) and clustering sequences using OptiClust [11] with an identity level of either 97% (mothur_97% pipeline) or 99% (mothur_99% pipeline). Clustering with different identity levels was the only step in the workflow, where the two mothur pipelines differed from one other. Finally, the consensus taxonomy for each OTU was determined with the *classify.otu* command.

### Statistical analysis

Statistical analyses and graphical outputs were conducted using the *microeco* [35] and *phyloseq* [36] packages in R [37]. Contaminant OTUs/ASVs were identified and removed by comparing sample data with that of extraction and amplification controls using the *decontam* package [38]. Rare OTUs/ASVs were removed based on a relative abundance threshold (pipeline-specific), which was applied sample-wise to account for different sequencing depths among libraries. The threshold was defined based on the relative account of singletons and doubletons among the libraries and calculated as follows: first, the mean read count per library was calculated and then, the proportion of 3 reads relative to this mean read count was determined to establish the threshold. This threshold was applied to each library independently, setting all OTUs/ASVs with a relative abundance below this threshold to zero. We calculated the threshold separately for each pipeline due to variations in the mean read count per library among pipelines (threshold for dada2: 0.0236%, threshold for both mothur pipelines: 0.0156%). On average, single libraries contained 12,700 (dada2) and 19,200 reads (both mothur pipelines). Removing OTUs/ASVs with a read count below 3 in one sample corresponds to excluding those with a relative abundance below a pipeline-specific threshold of 0.0236% (dada2) and 0.0156% for both mothur pipelines. After applying these relative abundance thresholds to our OTU/ASV-tables, the minimum read counts in libraries ranged from 2 reads (in libraries exhibiting low sequencing depth) to 9 reads (in libraries exhibiting high sequencing depth). On average, the minimum read count per sample was 3.59 for dada2 and 3.54 for both mothur pipelines.

Outputs of the three pipelines (dada2, mothur_97%, mothur_99%) were merged into one R object and compared. Four criteria were used to evaluate pipeline performance: (i) among biological replicates the proportion of the fungal community (as measured by alpha and beta diversity) captured by each pipeline was evaluated; among technical replicates we examined (ii) the proportion of OTUs/ASVs that were classified at each taxonomic level, (iii) the homogeneity of relative abundances of the most abundant genera between replicates and (iv) the capacity of each pipeline to detect OTUs/ASVs across replicates. To estimate alpha diversity in biological replicates, the number of observed OTUs/ASVs (richness), as well as Shannon and Simpson indices for bovine feces and soil samples using the *microeco* package [35] were determined. Alpha diversity was compared between pipelines within one sample type and between sample types within one pipeline using Duncan’s multiple range test for one-way ANOVA; *p*-values were adjusted using the Benjamini & Hochberg correction [39]. Beta diversity in biological replicates was represented by NMDS-ordination plots based on Bray-Curtis dissimilarities between samples. Significant differences between fungal communities of sample types and sampling sites were identified by applying a PERMANOVA (999 permutations). The *betadisper* function of the *vegan* package [40] was used to estimate the multivariate homogeneity of group dispersions (variances) and differences between sample types were examined with an ANOVA.

Proportions of classified OTUs/ASVs to unclassified OTUs/ASVs at phylum and genus level were calculated for each sample type and as means across technical replicates (*n* = 18). Significant effects on the relative abundance of the top 15 most abundant genera associated with pipelines were identified with a GLM and a Kruskal Wallis one-way ANOVA using the *ALDEx2* package [41–43], which takes the compositionality of barcoding data into account. Heterogeneity within one pipeline was calculated as follows: first, the mean and standard deviation of the relative abundance of the top 15 most abundant genera among all technical replicates (*n* = 18) per sample type was calculated. Then, the proportions of standard deviation to mean relative abundance (= coefficient of variation) were calculated for every genus. The mean of all proportions was considered as an index assessing pipeline heterogeneity among technical replicates and compared between pipelines.

A stepwise addition of OTUs/ASVs found in individual technical replicates (replicate no. 1 to no. 18, for each sample type) was calculated and these cumulative OTU/ASV numbers were represented as a line plot. The number of private OTUs/ASVs, i.e. those detected exclusively in individual replicates, was compared among pipelines. Rank abundance curves were calculated to visualize the evenness of OTU/ASV abundances among the different pipelines, using the *BiodiversityR* package [44].

## Results

### Differences in fungal communities estimated by the three pipelines

**Fig. 2:**
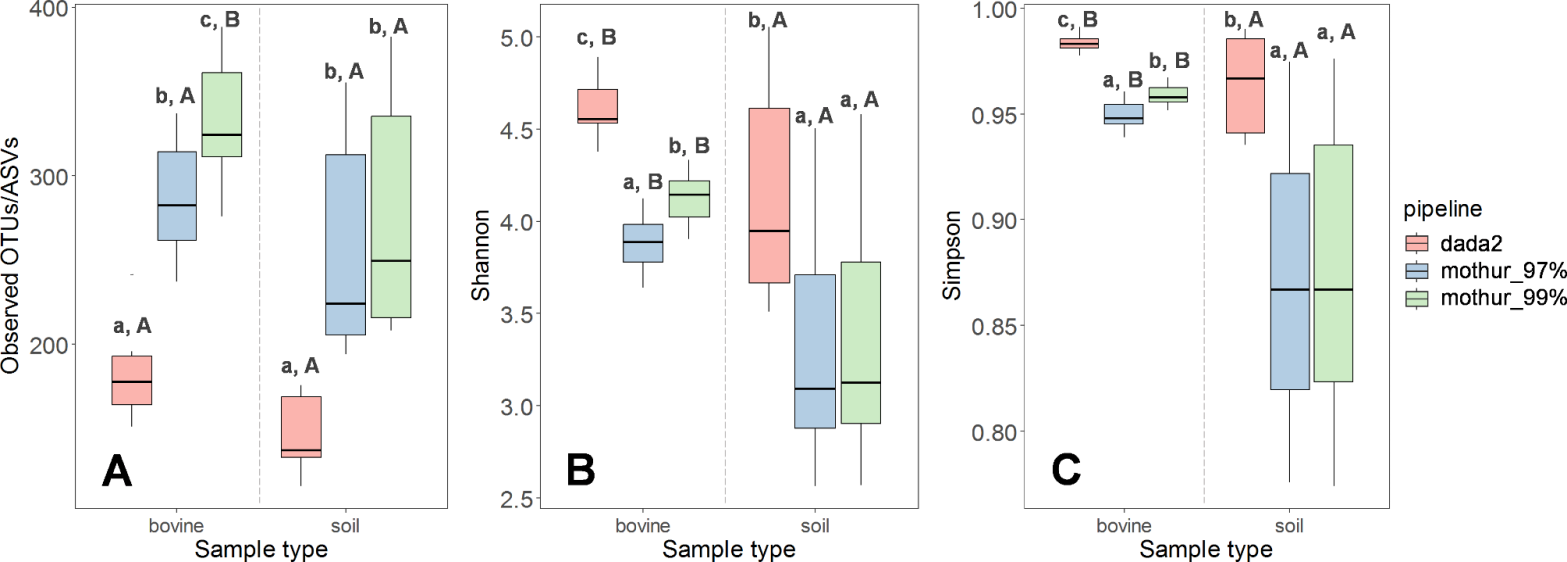
Box plots showing the alpha diversity measures Observed OTUs/ASVs (richness) (**A**), Shannon index (**B**) and Simpson index (**C**) of bovine feces (*n* = 10) and soil biological replicates (*n* = 9) across three metabarcoding pipelines. Lower-case letters indicate significant differences between pipelines within a sample type, while upper case letters indicate significant differences between sample types within one pipeline

The alpha and beta diversity of the fungal communities identified by the three pipelines was compared in a set of biological replicates to assess the similarity of pipelines outcomes. For both sample types, the dada2 pipeline identified a significantly lower absolute number of ASVs (richness) and had significantly higher estimates of both Shannon and Simpson indices compared to the mothur_97% and mothur_99% pipelines (Fig. 2). The mothur_97% pipeline generated significantly lower alpha diversity measures than mothur_99% in bovine fecal samples. In soil samples, the alpha diversity measures followed the same trend, although differences between pipelines were not significant. All pipelines consistently identified higher numbers of observed OTUs/ASVs (only mothur_99% significantly so), Shannon and Simpson indices (all three pipelines) in bovine feces samples compared with soil (Fig. 2). Notably, the alpha diversity outcomes were not consistent between pipelines when choosing pipeline-specific default settings (quality thresholds and bootstrapping cut-off) during sequence processing; e.g. Shannon measures were significantly higher in soil compared to bovine feces with dada2, while the opposite was the case with mothur_99% (see Supplementary Information, Supporting Results & Discussion section, Figure S3).

The fungal community compositions of bovine feces and soil samples were significantly different (PERMANOVA, *p* < 0.001), and consistently, no differences between sampling sites were found for each sample type for each pipeline (PERMANOVA, *p* > 0.05; Figure S1 A). However, beta diversity varied significantly with pipeline (PERMANOVA, *p* < 0.001). NMDS-plots based on Bray-Curtis dissimilarities showed that the sample clustering was similar for both mothur pipelines, whereas the sample distribution using dada2 was different. The analysis of multivariate homogeneity of group variances revealed that all pipelines consistently identified significant differences between bovine feces and soil samples. In both sample types, dada2 had the highest distances between samples and group centres (centroids) and mothur_97% the lowest (Figure S1 B).

## Pipeline-associated differences in community assembly and taxonomic classification in technical replicates

Sequencing yielded a total of 479,936 reads in bovine feces and 506,300 reads in soil technical replicates. After processing through the three pipelines, the basic read outputs were compared (Table 1). Homogeneity of sequencing depths was checked via rarefaction curve calculation (Figure S5). After processing, both mothur pipelines retained a higher total number of reads per sample type and mean per replicate, whereas dada2 retained a lower number of total and mean reads (Table 1). Likewise, the number of observed OTUs/ASVs (total per sample type and mean per replicate) was higher for mothur pipelines (highest with mothur_99%) than dada2. The removal of rare OTUs/ASVs also impacted the pipelines differently, causing a ~ 5% loss of OTUs/ASVs with dada2, ~ 57% loss with mothur_97% and ~ 79% loss with mothur_99%. Due to this step, the differences in observed OTUs/ASVs between pipelines was reduced: on average dada2 retained 681 ASVs for both sample types, mothur_97% 641 OTUs and mothur_99% kept 817 OTUs (Table 1). Dada2 showed the highest proportion of classified phyla and genera in individual replicates, followed by mothur_99% and mothur_97% (Table 1). The discrepancy between the sum and mean number of OTUs/ASVs obtained in the 18 replicates in each sample type differed across pipelines; for example, with dada2, the sum of all OTUs/ASVs was about five times higher than the mean number of OTUs/ASVs per individual replicate, whereas it was only three times higher for both mothur pipelines (Table 1). Of note, an additional analysis with dada2 revealed that pooling all technical replicates before the ASV construction resulted in 20% fewer observed ASVs (in total) than pooling all replicates following ASV inferring.

**Fig. 3:**
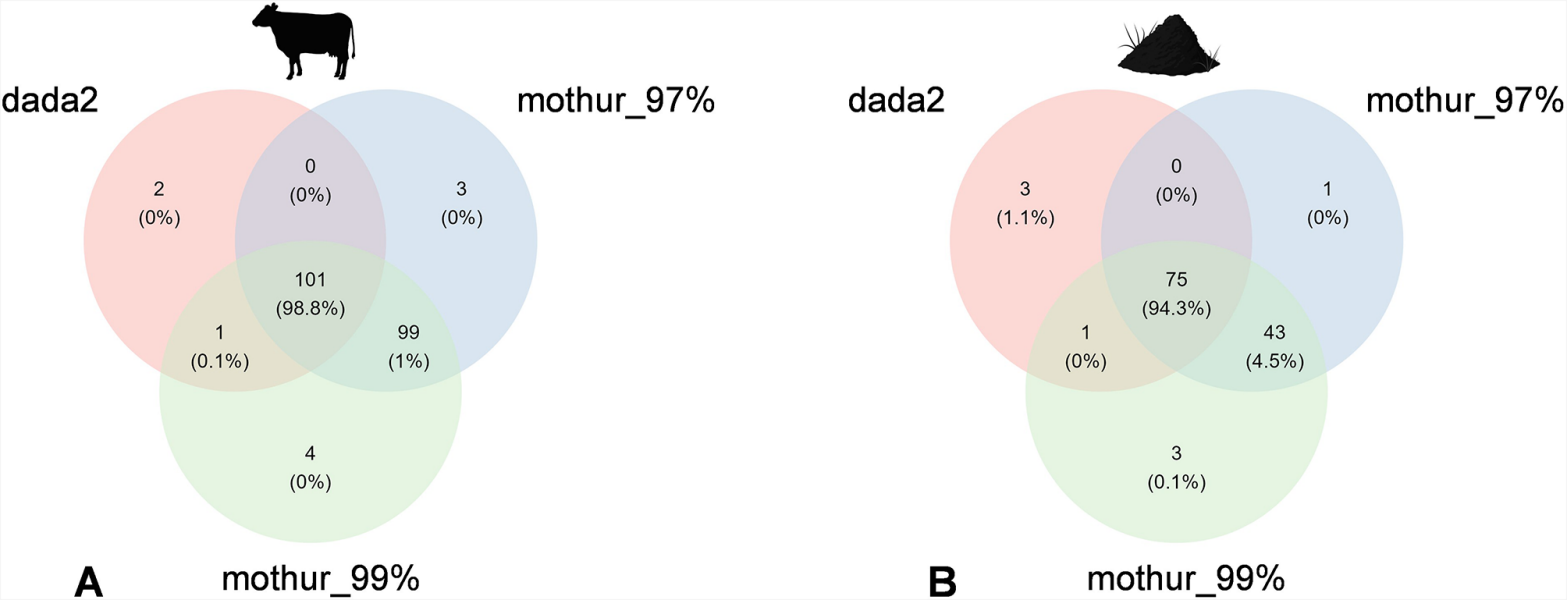
Venn diagrams representing the absolute number of shared and unique classified genera among different pipelines (dada2, mothur_97%, mothur_99%) in bovine feces (**A**) and soil (**B**) technical replicates. Values in brackets give the percentage of reads for the respective genera, expressed as a rounded proportion of the total reads

The identification of genera that were either commonly or exclusively detected by each pipeline (Fig. 3) was performed following the removal of all OTUs/ASVs not classified at least to genus level. In bovine feces replicates, 101 genera (accounting for 98.9% of all reads) were detected by all pipelines, whereas 2–4 genera were uniquely identified by only one pipeline (Fig. 3A). All of these uniquely identified genera had low read counts (max. 20 reads per genus; Table S1). Generally, the overlap of identified genera between mothur_97% and mothur_99% was higher than the overlap between dada2 and mothur pipelines. In soil replicates, 75 genera (accounting for 94.3% of all reads) were identified by all pipelines and 1–3 genera were uniquely identified by only one pipeline (Fig. 3B). Among these unique genera, generally exhibiting low read counts (maximum 12 reads), were two genera with high read counts (dada2: *Calycina* 6114 reads; mothur_99%: *Preussia* 647 reads; Table S1).

**Table 1:** Basic read output after processing input data with different pipelines (dada2, mothur_97%, mothur_99%) separately for different sample types (bovine feces, soil)

	bovine feces			soil		
	dada2	mothur_97%	mothur_99%	dada2	mothur_97%	mothur_99%
*Input*						
Number of reads	479 936	479 936	479 936	506 030	506 030	506 030
*Post-processing*						
Number of reads	186 019	284 714	284 718	215 200	317 107	317 104
Total number of OTUs/ASVs	714	1 499	4 293	718	1 512	3 317
Mean number of OTUs/ASVs / replicate	126	368	622	131	439	582
Mean library size	10 334.39	15 817.44	15 817.67	11 955.56	17 617.06	17 616.89
Minimum library size	6 356	10 335	10 335	7 173	11 118	11 118
Maximum library size	14 079	21 227	21 228	23 671	29 762	29 761
*Following sample-wise removal of rare OTUs/ASVs*						
Number of reads	185 974	281 135	276 803	214 988	311 475	308 826
Total number of OTUs/ASVs	697	751	1 019	665	531	616
Mean number of OTUs/ASVs / replicate	124	206	252	127	208	221
Mean library size	10 331.89	15 618.61	15 377.94	11 943.77	17 304.17	17 157
Minimum library size	6 356	10 247	10 100	7 173	10 973	10 875
Maximum library size	14 071	20 926	20 625	23 559	29 203	28 994
Mean number of OTUs/ASVs classified to phylum level / sample	119	194	230	117	189	197
Mean number of OTUs/ASVs classified to phylum level / sample [%]	96	87	88	92	86	87
Mean number of OTUs/ASVs classified to genus level / sample	83	129	152	78	108	114
Mean number of OTUs/ASVs classified to genus level / sample [%]	67	58	58	61	50	50
Number of classified genera	104	203	205	79	119	122
Mean number of classified genera / replicate	33	84	83	36	68	68

### Homogeneity of relative abundances among pipelines and OTU/ASV detection

**Fig. 4:**
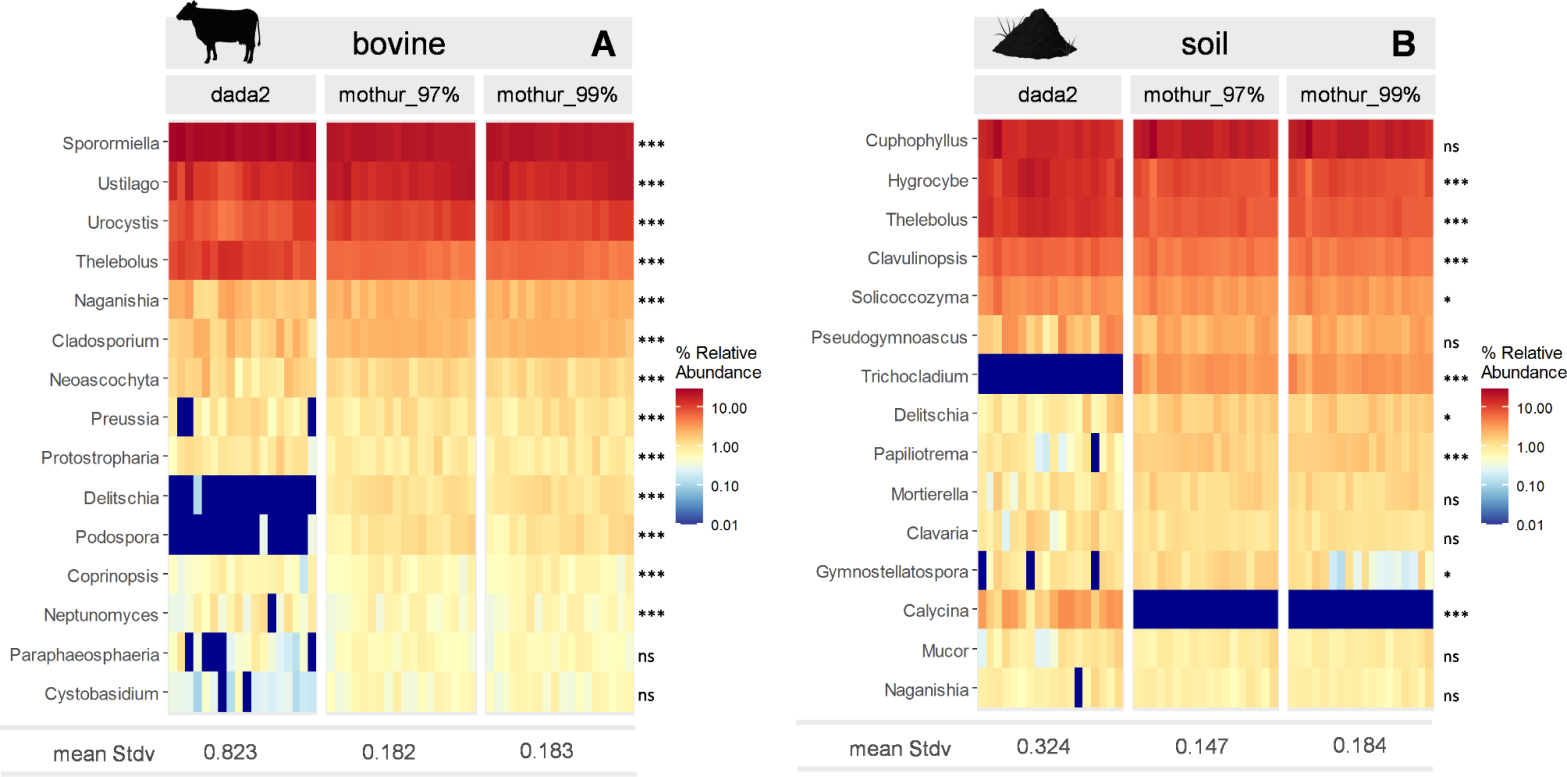
Heatmaps for bovine feces (**A**) and soil (**B**) technical replicates showing the relative abundance of 15 most abundant fungal genera, identified by different pipelines (dada2, mothur_97%, mothur_99%). Significant differences among pipelines are indicated by asterisks (*p* < 0.001 ***, *p* < 0.01 **, *p* < 0.05 *, *p* > 0.05 ns). The mean ratio of standard deviation to mean relative abundance (mean Stdv) for every pipeline is given below the heatmaps

Structural community composition was compared using the 15 most abundant genera among all pipelines in technical replicates (bovine feces and soil, respectively; Fig. 4). Pipelines provided contrasting results regarding relative abundances of these selected genera for both sample types. In bovine feces, 13 out of 15 genera had significantly different relative abundances (*p* < 0.001) among the tested pipelines. In soil samples, 9 out of 15 genera showed significantly different abundances (*p* < 0.05) depending on the pipeline, e.g. dada2 failed to identify the genus *Trichocladium*; while mothur pipelines missed the genus *Calycina*, both genera with high abundance according to the opposite software (Fig. 4). While the genus *Calycina* was as least represented on a higher taxonomic level in mothur (e.g. family Hyaloscyphaceae, consistently found to be highly abundant by all pipelines), the genus *Trichocladium* was underrepresented by dada2 even at higher taxonomic levels (e.g. family Chaetomiaceae: 67 reads, sum of all samples, abundance estimated by dada2). Importantly, dada2 showed a significantly higher heterogeneity between the technical replicates (ANOVA, *p* < 0.005), than mothur_99% and mothur_97% (Fig. 4).

**Fig. 5:**
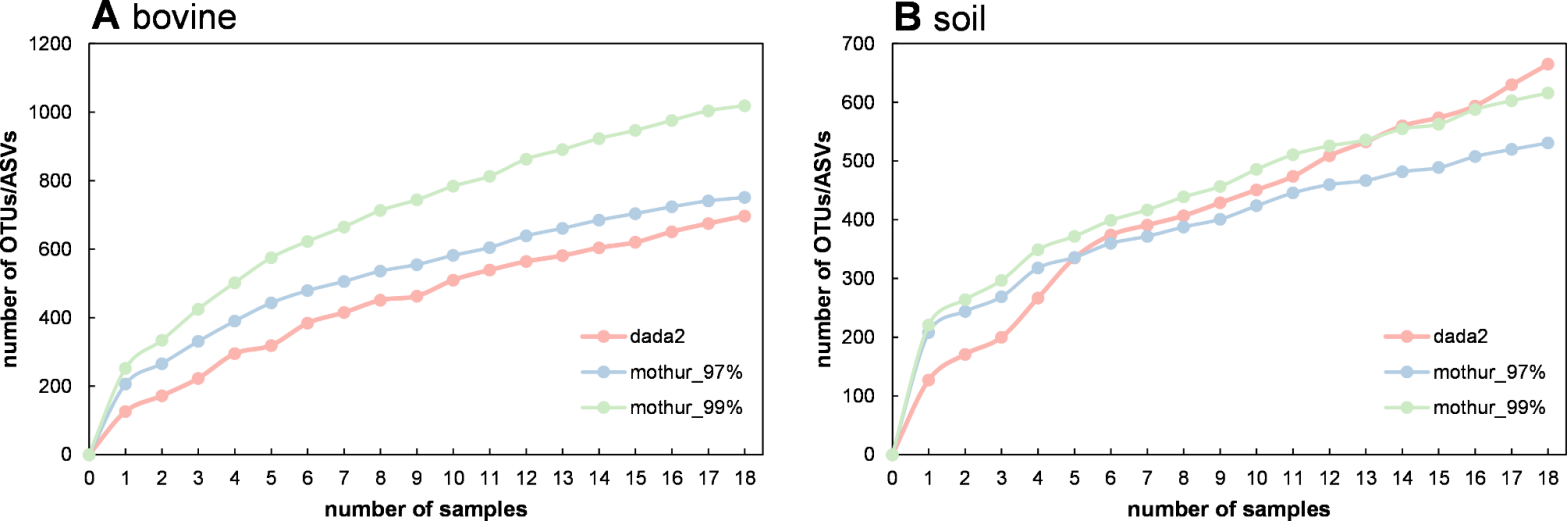
Additive number of OTUs/ASVs in bovine feces (**A**) and soil (**B**) based on the number of technical replicates from three different analysis pipelines: dada2, mothur_97%, mothur_99%. Identified OTUs/ASVs are shown as cumulative numbers for 1–18 technical replicates, whereby private OTUs/ASVs are added stepwise to the total number of OTUs/ASVs

A stepwise addition of OTUs/ASVs in technical replicates showed that dada2 and mothur_97% had a similar rate of increase of OTUs/ASVs in bovine feces samples, while mothur_99% showed a steeper increase. However, in soil samples, OTU/ASV numbers of both mothur pipelines (especially mothur_97%) plateaued, while the numbers found with dada2 showed a continuous steep increase (Fig. 5). Importantly, we found that by reducing the sample size from 18 to three technical replicates (a number of replicates more commonly used in microbiota studies), dada2 only detected about 31% of all OTUs/ASVs (i.e. sum of 18 technical replicates for bovine feces and soil samples, respectively) compared with mothur_97% and mothur_99%, which detected about 43% of all OTUs/ASVs (sum of 18 technical replicates, mean of both mothur pipelines) in bovine feces samples, and almost 50% of all OTUs/ASVs (sum of 18 technical replicates, mean of both mothur pipelines) in soil samples (Fig. 5A, B). Of note, after processing our data with non-unified and specifically recommended quality thresholds for each pipeline (see Supplementary Information, Material & Methods section), stepwise addition of OTUs/ASVs in technical replicates showed that the number of OTUs/ASVs plateaued for mothur pipelines at a sample size of ± 10 samples, whereas dada2 showed an almost linear increase of OTU/ASV numbers (see Supplementary Information, Results & Discussion section, Figure S4); at a sample size of three, dada2 detected 16.7% of all OTUs/ASVs, whereas mothur_97% and mothur_99% identified up to 66.7% of all OTUs/ASVs.

Rank abundance curves of the top 50 most abundant OTUs/ASVs demonstrated that the evenness among the highly abundant OTUs/ASVs was higher with dada2 (moderate decrease of the line) than with both mothur pipelines (steep decrease of the line), and this was more evident for soil (Supplementary Information, Results & Discussion section, Figure S2 A, B).

## Discussion

Metabarcoding is now an indispensable tool for community studies, but the bioinformatic analysis of the resulting large datasets is challenging; therefore, having practical guidelines available for choosing the appropriate pipeline(s) can save valuable time. In this study, we compared the OTU-clustering approach in mothur, using two identity thresholds, with the ASV-inferring method in DADA2 with metabarcoding data from the ITS2 region in fungal communities from bovine feces and soil samples. In detail, we compared the fungal output of three pipelines, which we named: dada2, mothur_97% and mothur_99%. They have been developed and commonly used for analyzing prokaryote (especially bacterial) amplicon sequences: while DADA2 software has been increasingly applied to fungal communities, mothur (either with 97 or 99% identity) has received relatively less attention. Our aim was to evaluate if and how these bioinformatic strategies impact fungal community diversity and composition in environmental samples. We processed 19 biological replicates (10 bovine feces and 9 soil replicates) and 36 technical replicates (*n* = 18 replicates of one bovine feces and one soil sample) with the three different pipelines, and used four criteria to evaluate pipeline performance (specified in Materials and methods section). In general, although outputs of the three pipelines were significantly different for some indices, the conclusions (e.g. differences of alpha diversity between sample types) were consistent. However, processing sequencing data with pipeline-specific default quality thresholds and bootstrapping cut-offs which, to the best of our knowledge, is the most frequent analytical strategy, leads to opposing conclusions (see Supplementary Information, Material & Methods section; Figure S3).

### Proportion of the fungal community captured by the pipeline (alpha and beta diversity)

Some studies have suggested that ecological patterns of microbiota are quite robust regardless of the bioinformatic pipeline used to analyse amplicon datasets [45, 46]. This conclusion was confirmed in our study. We showed that the pipelines used here consistently identified significantly higher alpha diversity estimates in bovine feces compared with soil samples (albeit not significantly for dada2 and mothur_97% richness estimates). However, we also showed that the consistency among pipelines was due to the adoption of the same settings (quality thresholds and bootstrapping cut-off), instead of default settings, in each pipeline during sequence processing. By applying the same settings, within each sample type, the alpha diversity estimates differed substantially between the pipelines: dada2 exhibited the lowest species richness followed by mothur_97% and mothur_99%. Previous studies have also found such discrepancies between different sets of pipelines and suggested that absolute estimates of species richness should not be overinterpreted and that metabarcoding studies should focus on the differences between samples [19, 20, 47]. Overall, our comparison revealed homogeneity among pipelines regarding diversity conclusions. However, we showed that this homogeneity is disrupted by adopting default and/or recommended pipeline settings. This highlights the importance of carefully specifying settings for pipelines. Adaptations might have a great impact on the outcomes regarding fungal community analysis (e.g. species richness, OTU/ASV-detection).

Despite the similar conclusions drawn by the pipelines, their alpha diversity measures differed significantly. One of the most important differences between pipelines is the clustering or data-filtering approach generating OTUs or ASVs, already well-recognized as estimating different, pipeline-specific results for bacterial communities [48]. However, the discussion whether OTU clustering or ASV inference is better suited for fungal ITS metabarcoding data is ongoing [1, 7, 15]. Although ASV approaches were shown to recover mock communities of several fungal strains better than OTU clustering approaches [19, 49], they might overestimate fungal diversity when using markers with a high level of intraspecific variation (e.g. ITS2 subregion). Due to the high sensitivity of ASV approaches, allelic variants of the ITS region will be assigned to different ASVs and inflate the fungal diversity [16, 17]. On the other hand, ASV approaches likely underestimate the richness of less prevalent fungal species, due to removal of less abundant ASVs during ASV construction [7, 20]. In contrast, comparing mothur’s OTU clustering with ASV approaches for 16S rRNA amplicon data analysis have suggested that mothur tends to overestimates richness [48, 50]. Our findings indicate significantly lower fungal richness in bovine feces and soil samples processed with the ASV approach (dada2) compared with the OTU clustering methods (mothur_97%, mothur_99%). On the contrary, higher Shannon and Simpson indices were estimated for both sample types with dada2 compared with mothur pipelines. This confirms an overestimation of abundant ASVs with the ASV approach. In addition, rank abundance curves showed that dada2 identified a higher number of highly abundant ASVs than both mothur pipelines (OTUs), further confirming an overestimation of abundant ASVs (Figure S2).

In addition to the clustering or data-filtering approaches of the three pipelines, the mothur pipelines were applied here using two identity thresholds for OTU clustering, which also affected the alpha diversity measures. Fungal sequences are commonly clustered to OTUs based on 97% identity [7]; however, higher thresholds have been proposed as more appropriate for fungal data [51]. Here, we applied both 97% and 99% similarity thresholds and found significant differences across the corresponding fungal community in bovine feces (but not soil). In fact, mothur_97% resulted in lower numbers of observed OTUs (richness), as well as lower estimates for both Shannon and Simpson indices than mothur_99% among biological replicates. Lower diversity observed with mothur_97% may result from the collapsing of erroneous sequences or intragenomic variations into other / fewer OTUs, as well as from aggregating distinct species due to the 97% similarity threshold. On the other hand, a higher similarity threshold (e.g. 99%) could have retained more ‘true’ species [26, 52], but also OTUs that originate from intragenomic variation [53]. Due to intragenomic variations, multiple copies of the ITS region can occur within one species [54]. This heterogeneity complicates species identification with HTS approaches and might lead to an overestimation of species richness. This overestimation could be more severe when applying higher similarity thresholds, as more intragenomic variation will be incorrectly clustered into distinct OTUs [55, 56]; for example, in our environmental samples, clustering with 99% similarity (mothur_99%) resulted in more than twice as many OTUs as with 97% similarity (mothur_97%) (1499 and 4293 OTUs in bovine feces, 1512 and 3317 OTUs in soil samples using 97% and 99% similarity thresholds; including rare OTUs). Likewise, other studies found more fungal OTUs in environmental samples when applying similarity thresholds higher than 97% [13, 57]. We assume that an incorrect allocation of intragenomic variations to distinct OTUs was higher when using the higher similarity threshold (99%) in our analysis, and this could, combined with sequencing errors, lead to erroneously high richness results [58]. However, with the present sequencing data and available tools, we cannot confirm or correct these errors. Even if the ITS region is appropriate for species identification for a broad range of fungi, it is clearly not appropriate for all fungi due to their different rates of evolution [13, 59]. Increasing the taxonomic coverage of reference databases, performing large-scale species identifications and adapting existing bioinformatic pipelines might be solutions for dealing with intragenomic variations in future studies [16, 60].

In addition to intragenomic variation, PCR and sequencing errors possibly also result in the generation of rare (and false) OTUs [12, 16], and removing these invalid OTUs is recommended [61]. Although we minimized PCR errors by using a High Fidelity Polymerase with 3’-5’ proofreading activity, we removed rare OTUs/ASVs (using a relative abundance threshold, applied sample-wise) from our dataset to avoid overestimation of alpha diversity. We found that many OTUs (748 and 3274 OTUs in bovine feces, 981 and 2701 OTUs in soil samples using 97% and 99% similarity thresholds, respectively) were discarded by filtering these rare OTUs. With mothur_99%, filtering removed about 79% of all OTUs, which is a much higher percentage than for mothur_97% results (57% OTUs removed). This is probably due to the lower number of rare OTUs identified with mothur_97%, since applying lower similarity thresholds (here: 97% compared with 99% similarity) results in merging of rare OTUs with other low-abundance or abundant OTUs [62]. Overall, this filtering step converged the total numbers of OTUs/ASVs observed with different similarity thresholds (mothur_97% and mothur_99%) and clustering methods (dada2) (Table 1, total number of OTUs/ASVs in *Post-processing* section compared with *Following sample-wise removal of rare* *OTUs/ASVs* section). These results are in line with the findings of [63], where similar richness estimates among different pipelines were achieved by filtering of rare OTUs.

### Proportion of OTUs/ASVs that were classified to genus level

Although the same classifier (RDP Naive Bayesian Classifier algorithm) and the same minimum bootstrap confidence value (80% cutoff) was used for the taxonomic assignment for all pipelines, the ratio of identified phyla and genera in single technical replicates differed among the pipelines. Dada2 classified a higher proportion of OTUs/ASVs to phylum and genus level in single replicates compared with mothur pipelines; however, considering absolute numbers, dada2 identified a lower number of phyla and genera than both mothur pipelines, which is in line with the richness results of OTUs/ASVs among pipelines. Data processing with the pipelines mothur_97% and mothur_99% identified similar numbers of genera. In both sample types, about half of the identified genera (50% in bovine feces, 60% in soil) were detected by all pipelines, however those shared genera account for > 94% of all sequences found in bovine feces and soil samples, respectively. We conclude that, despite differences in their relative abundance estimates (see below), abundant genera were detected by all pipelines, and mainly rare genera, exhibiting only low read counts, were assigned to one specific pipeline. In fact, every pipeline identified a few unique genera with low read counts (2–20 reads). These could have possibly emerged from sequencing errors [1, 64] and a more stringent filtering of rare OTUs/ASVs would have eliminated most of these unique genera. Generally, taxonomic assignments at such high taxonomic resolution should not be overvalued, as taxonomic identification of OTUs/ASVs might be inadequate [65]. Nevertheless, dada2 and mothur_99% each identified one abundant genus (dada2: *Calycina* 6114 reads; mothur_99%: *Preussia* 647 reads) in soil, which both are saprotroph (-symbiotroph) and decay dung or wood [66]. Considering the trophic modes, we find it plausible that these genera were present in our soil samples, making it concerning that the pipelines missed them. However, lowering the taxonomic resolution to the family level revealed that all three pipelines identified the families to which these genera belong, but did not classify the associated OTUs further to the genus level. For future comparative studies, lowering the bootstrap threshold in taxonomic classification (losing reliability of the given results) could be considered to retain more fungal genera.

### Homogeneity of relative abundances and OTU/ASV detection in technical replicates

Coherently with the discussion above, in our comparison of community compositions among pipelines we focused here on the top 15 most abundant fungal genera in both bovine feces and soil technical replicates. We found that the majority of abundant genera exhibited significantly inconsistent proportions among different pipelines. Of particular note is that some genera (e.g. *Calycina*,* Delitschia*) were found to be highly abundant either in bovine feces and soil replicates by one pipeline (dada2 and mothur_97%), but were not even identified in most replicates by another pipeline (mothur_97% and dada2). We also found that the homogeneity of the relative abundances of most abundant genera among the replicates (*n* = 18) differed according to pipeline. Overall, dada2 exhibited a significantly higher heterogeneity in bovine feces and soil replicates than mothur pipelines (Fig. 4; mean Stdv), but mothur_97% showed the least heterogeneity. As our samples consisted of technical replicates (*n* = 18) of one sample per environmental sample type (bovine feces and soil), and theoretically fungal community composition should be identical, we conclude that the pipeline with the highest homogeneity among all technical replicates (mothur_97%) would be the best suited to describe a fungal community and also – in comparable studies – to identify differences between different samples due to a lower intern variability of technical replicates.

We also explored the variability in the number of private OTUs/ASVs (i.e. those found exclusively in individual replicates) among different pipelines. Results indicated that pipelines varied in their capacity to detect OTUs/ASVs in different sample types and depending on sample number. While both mothur pipelines (mothur_97%, mothur_99%) detected 31.8% of all possible OTUs for bovine feces or soil in a single replicate, dada2 only detected 18.5% of all ASVs per replicate (see Fig. 5A, B). This means that if the number of replicates per sample type was lowered to that similar to a field experiment with many sites (e.g. three replicates) only 32% of all ASVs (18 replicates) would be identified with dada2, whereas mothur_97% and mothur_99% would identify 46.2% of all OTUs (see Fig. 5). This discrepancy is attributed to the distinct patterns observed in cumulative taxonomic numbers. The mothur pipelines, particularly mothur_97% for soil replicates, demonstrated a more efficient OTU/ASV detection with fewer replicates, which is advantageous. Notably, applying pipeline-specific recommendations for quality filtering and bootstrap cut-offs during sequence processing led to a plateau in OTU detection during stepwise addition of OTUs in case of the mothur pipelines, demonstrating sufficient detection even with fewer replicates. In contrast, dada2 showed an almost linear increase in ASVs, with a high number of private ASVs. The analysis of private OTUs/ASVs indicates that the mothur pipelines exhibit better OTU/ASV detection than dada2, and that mothur’s default settings during sequence processing result in sufficient OTU/ASV detection among technical replicates.

## Conclusions

Overall, our study highlights the impact of bioinformatic pipeline selection on fungal metabarcoding data. The comparison revealed significant differences in the results obtained from commonly used pipelines, particularly when pipeline-specific default or recommended settings are used. We found that species richness in biological replicates was significantly higher in mothur pipelines (highest with mothur_99%) compared with dada2. The dada2 pipeline (ASV approach) showed the greatest heterogeneity of relative abundances and a poorer OTU/ASV detection across technical replicates (*n* = 18) compared with mothur pipelines. In summary, we want to (i) generally draw attention to the great impact of pipeline settings on sufficient OTU/ASV detection and (ii) point out that the OTU approach outcompeted the ASV approach, due to a more efficient OTU detection and a great homogeneity among technical replicates. Hence, we recommend using a pipeline with OTU clustering (e.g. mothur_97%) and a careful reflection of respective pipeline settings for future studies.

## Electronic supplementary material

Below is the link to the electronic supplementary material.

Supplementary Material 1

Supplementary Material 2

## Data Availability

The datasets analyzed during the current study are available from the corresponding author upon reasonable request. Raw sequence reads are deposited in the SRA (BioProject PRJNA1055419).
