# Supplementary material for: Comparison of commonly used software pipelines for analyzing fungal metabarcoding data
Source: BMC Genomics. 2024 Nov 14;25:1085. doi: 10.1186/s12864-024-11001-x (PMC11566164; doi:10.1186/s12864-024-11001-x)
Supplement: Supplementary file 1 — Supplementary Material 1 [file 12864_2024_11001_MOESM1_ESM.docx]

**Comparison of commonly used software pipelines for analyzing fungal metabarcoding data**

Theresa Rzehak^1*^, Nadine Praeg^1^, Giulio Galla^2^, Julia Seeber^3,4^, Heidi C. Hauffe^2,5^, Paul Illmer^1^

^1^ Department of Microbiology, Universität Innsbruck, Innsbruck, Austria

^2^ Conservation Genomics Research Unit, Research and Innovation Centre, Fondazione Edmund Mach, S. Michele all'Adige, Italy

^3^ Institute for Alpine Environment, EURAC Research, Bozen, Italy

^4^ Department of Ecology, Universität Innsbruck, Innsbruck, Austria

^5^National Biodiversity Future Center (NBFC), S.c.a.r.l., Palermo, Italy

*corresponding author

**Supplementary Material**

**Supporting Material & Methods**

1. **Analysis with unified settings for quality filtering and taxonomic assignment**

*1.1 Bioinformatic downstream analysis generating ASVs – dada2 pipeline*

Barcode free, paired-end reads of demultiplexed samples were processed following an ITS-specific adaptation of the 1.8 DADA2 tutorial workflow (<https://benjjneb.github.io/dada2/ITS_workflow.html>), using the DADA2 package (Callahan et al. 2016) in R (v 4.2.0, R Core Team 2022) (hereafter ‘dada2 pipeline’). Primers were removed with Cutadapt. Then, reads were quality filtered using the *filterAndtrim* function. Reads less than 50 bp in length, having ambiguous bases or ‘bad quality’ were discarded, where bad quality reads were defined as reads not passing the *filterAndtrim*-parameters truncQ = 8, maxEE = 6. We increased the values of these filtering parameters (default values are truncQ = 2, maxEE = c(2, 2), according to the recommendations of Rolling et al. (2022).

*1.2 Bioinformatic downstream analysis generating OTUs – mothur_97% and mothur_99% pipelines*

Fungal OTUs were constructed using mothur (v.1.48.0) following the MiSeq SOP (last access 6/10/22) (Schloss et al. 2009). Forward and reverse reads were merged using the *make.contigs* function with default filtering parameters (insert = 20, deltaq = 6). After primers were trimmed with the *trim.seq* function, sequences with less than 100 bp in length were discarded.

**Supporting Results & Discussion**

**Table S1:** Classified fungal genera in bovine feces (left) feces and soil (right) that were uniquely identified by each pipeline (dada2, mothur_97%, mothur_99%) and total number of reads per genus. In total, 306 different, classified genera were found (157 genera with dada2, 266 with mothur_97% and 271 with mothur_99%).

|  | **bovine feces** | | **soil** | |
| --- | --- | --- | --- | --- |
|  | Genus | total reads | Genus | total reads |
| **dada2** | *Clitocybe* | 9 | *Calycina* | 6114 |
|  | *Hypholoma* | 3 | *Calycellina* | 6 |
|  |  |  | *Lachnellula* | 2 |
|  |  |  |  |  |
| **mothur_97%** | *Pseudeurotium* | 20 | *Clarireedia* | 10 |
|  | *Glarea* | 3 |  |  |
|  | *Mycenastrum* | 3 |  |  |
|  |  |  |  |  |
| **mothur_99%** | *Septoriella* | 3 | *Preussia* | 647 |
|  | *Cleistothelebolus* | 17 | *Geomyces* | 12 |
|  | *Cadophora* | 6 | *Trimmatostroma* | 4 |
|  | *Myrmecridium* | 2 |  |  |
|  |  |  |  |  |

**Figure S1:** NMDS-ordination plots based on Bray-Curtis dissimilarities (**A**) and box plots of distances to group centroids (**B**) showing biological replicate samples of bovine feces (n = 10) and soil samples (n = 9) separately for three metabarcoding pipelines: dada2, mothur_97%, mothur_99%; Sample types are represented by different colors and sampling sites by different shapes; letters indicate significant differences between sample types;

**Figure S2:** Rank abundance curves of top 50 OTUs/ASVs (rank) in technical replicates of bovine feces (**A**) and soil (**B**). Abundance of OTUs/ASVs was log transformed (log_10_ abundance). Different colors represent different analysis pipelines: dada2, mothur_97%, mothur_99%.

Observed OTUs/ASVs

**Figure S3:** Box plots showing the alpha diversity measures Observed OTUs/ASVs (**A**), Shannon index (**B**) and Simpson index (**C**) of bovine feces (n = 18) and soil samples (n = 18) across three metabarcoding pipelines. Notably, the number of replicates (n) denotes technical replicates. Lower-case letters indicate significant differences between pipelines within a sample type, while upper case letters indicate significant differences between sample types within one pipeline (Duncan’s multiple range test, *p* < 0.05).

By following the pipelines’ recommendations, the tested pipelines used for analysing the fungal communities did not result in similar biological conclusions: although the pipelines consistently identified a higher number of observed OTUs/ASVs in soil compared to bovine feces (but mothur_99% was not significant), they resulted in contrasting results of Shannon and Simpson diversities for the two sample types: mothur_97% detected no significant difference, mothur_99% detected a significantly higher diversity in bovine feces samples, whereas the contrary was detected with dada2.

While the number of observed OTUs/ASVs is often biased due to low-abundance OTUs, measures that weight rare and abundant OTUs differently (e.g. Shannon and Simpson indices) are considered less biased by rare OTUs and hence more consistent (Bálint et al. 2016). Nevertheless, we confirmed that all applied alpha diversity measures (Observed OTUs/ASVs, Shannon and Simpson indices) were driven by the pipeline applied and that pipelines estimate the diversity among our sample types differently.

**Figure S4:** Additive number of OTUs/ASVs in bovine feces (**A**) and soil (**B**) based on the number of technical replicates from three different analysis pipelines: dada2, mothur_97%, mothur_99%. Pipelines were applied using non-unified and pipeline specifically recommended quality thresholds. Identified OTUs/ASVs are shown as cumulative numbers for 1–18 technical replicates, whereby private OTUs/ASVs are added stepwise to the total number of OTUs/ASVs. Horizontal lines mark the total sum of OTUs/ASVs (18 replicate samples) for each pipeline.

Following the recommended protocols for fungal analysis (see Supplementary Material & Methods), a stepwise addition of OTUs/ASVs found in replicate samples revealed distinct patterns. For the mothur pipelines, the number of OTUs/ASVs reached a plateau at approximately 10 samples. In contrast, dada2 continued to add private OTUs/ASVs, those detected exclusively in individual replicates, resulting in an almost linear increase in the number of OTUs/ASVs (Figure S4). While both mothur pipelines (mothur_97%, mothur_99%) detected about half of all possible OTUs/ASVs for bovine feces or soil in a single replicate, dada2 only detected 16.7% of all OTUs/ASVs per replicate (see Figure S4 A, B). This means that if the number of replicates per sample type was lowered to that similar to a field experiment with many sites (e.g. three replicates) only 33.3% of all OTUs/ASVs (18 replicates) would be identified with dada2, whereas mothur_97% and mothur_99% would identify 66.7% of all OTUs/ASVs (see Figure S4). This discrepancy is attributed to the distinct patterns observed in cumulative taxonomic numbers, where dada2 exhibits a linear increase in the number of OTUs/ASVs with each additional replicate due to high numbers of private OTUs/ASVs in replicates, whereas mothur pipelines reached a plateau, indicating a more efficient OTU detection with a lower number of replicates (Figure S4).

**Figure S5:** Rarefaction curves for technical replicates (n=18) showing the number of observed OTUs/ASVs per sample size obtained with three pipelines: dada2 (**A**, **B**), mothur_97% (**C**, **D**), mothur_99% (**E**, **F**). Different sample types are represented by different colors (bovine feces (**A**, **C**, **E**), bulk soil (**B**, **D**, **F**)). The rarefaction curves are labeled with replicate numbers referring to the sample table (see supplement).

**References**

Rolling, Thierry; Zhai, Bing; Frame, John; Hohl, Tobias M.; Taur, Ying (2022): Customization of a DADA2-based pipeline for fungal internal transcribed spacer 1 (ITS1) amplicon data sets. In *JCI insight* 7 (1). DOI: 10.1172/jci.insight.151663.

Schloss, Patrick D.; Westcott, Sarah L.; Ryabin, Thomas; Hall, Justine R.; Hartmann, Martin; Hollister, Emily B. et al. (2009): Introducing mothur: open-source, platform-independent, community-supported software for describing and comparing microbial communities. In *Applied and environmental microbiology* 75 (23), pp. 7537–7541. DOI: 10.1128/AEM.01541-09.
